# Esomeprazole and apixaban pharmacokinetic interactions in healthy rats

**DOI:** 10.1016/j.heliyon.2022.e11015

**Published:** 2022-10-12

**Authors:** Ali Jaber, Israa Al-Ani, Mohammad Hailat, Enas Daoud, Anmar Abu-Rumman, Zainab Zakaraya, Bashar J.M. Majeed, Osaid Al Meanazel, Wael Abu Dayyih

**Affiliations:** a Faculty of Pharmacy, Al-Ahliyya Amman University, Jordan; b Pharmacological and Diagnostic Research Center (PDRC) in Al-Ahliyya Amman University, Jordan; c Faculty of Pharmacy, Al-Zaytoonah University of Jordan, Amman 11733, Jordan; d Prince Hussain Hospital, Ministry of Health, Jordan; e Michael Sayegh Faculty of Pharmacy, Aqaba University of Technology, Aqaba, Jordan; f Faculty of Pharmacy, Mutah University, Jordan

**Keywords:** Apixaban, Esomeprazole, Mass spectroscopy, Drug interactions, Non-compartmental analysis

## Abstract

Esomeprazole is used in various clinical settings where a decrease in gastric acid production is desired since it is a proton pump inhibitor. Apixaban, an anticoagulant, is used to reduce the risk of stroke in patients with certain cardiovascular diseases. This research aims to examine the effects of giving esomeprazole and apixaban to rats simultaneously, as well as to measure their pharmacokinetics and look for statistical differences or interactions. A method for the simultaneous determination of esomeprazole and apixaban in rat plasma was developed using HPLC/MS and validated by ICH guidelines. Five groups of Wistar rats were created, and the drugs were administered as follows: esomeprazole (5 mg/kg) intravenously, apixaban (125 mcg/Kg) intravenously, esomeprazole (5 mg/kg) orally, apixaban (250 mcg/kg) orally, and esomeprazole (5 mg/kg) and apixaban (250 mcg/kg) both orally. Both drugs' concentrations were measured in plasma samples collected on a predetermined schedule. The pharmacokinetics of both drugs were calculated and statistically analyzed using a 90% confidence interval and non-compartmental analysis. When the two drugs were combined, apixaban’s C_max_ and AUC increased while esomeprazole’s C_max_ and AUC decreased. On the other hand, Apixaban’s T_max_ decreased with an increase in esomeprazole’s T_max_, indicating a possible interaction between the two drugs. When both drugs were taken together, their bioavailability was reduced, implying that less esomeprazole was absorbed over time.

## 1 Introduction

In general, drug-drug interactions are not uncommon among patients [1]. Esomeprazole (ESO), a proton pump inhibitor (PPI), is a 5-methoxy-2-{[(4-methoxy-3,5-dimethylpyridin-2-yl)methyl]sulfinyl}-1H-benzimidazole that has an *S* configuration at the sulfur atom [2]. It is a gastric acid secretion inhibitor used to treat gastroesophageal reflux, dyspepsia, peptic ulcer disease, and Zollinger-Ellison syndrome (generally sodium or magnesium salt) [3, 4]. It is the *S*-isomer of omeprazole, with gastric proton pump inhibitor activity. In the acidic compartment of parietal cells, ESO is protonated and converted into the active achiral sulfenamide. The active sulfenamide forms covalent disulfide bonds with the proton pump hydrogen-potassium adenosine triphosphatase (H^+^/K^+^ ATPase), thereby inhibiting its activity of parietal cell secretion of H^+^ ions into the gastric lumen, the final step in gastric acid production. H^+^/K^+^ ATPase is an integral membrane protein of the gastric parietal cell [3, 5]. Studies have shown that the absorption of ESO occurs in the small intestine and peak plasma concentration (C_max_) occurs in 1.5 h (T_max_), and that absorption is highly impaired by food intake [2]. After 20 or 40 mg delayed-release tablets, bioavailability is about 90%, and the mean exposure AUC increases with continuous use. It has a volume of distribution of about 16–18 L in adults and plasma protein binding of about 97% mainly to albumin, which is clinically affected in hypoalbuminemia [6, 7]. ESO is metabolized in the liver via Cytochrome 2C19 (CYP2C19) to hydroxy and desmethyl metabolites. Another minor pathway through CYP3A4 metabolized ESO to sulphone derivative [8].

Apixaban (AP) is an oral inhibitor of factor ten (FXa) or called the “coagulation factor” or “thrombin”. It binds and inhibits free and clot-bound factors and prothrombinase activity [9]. It does not require antithrombin III for antithrombotic activity. AP does not directly affect platelet aggregation but indirectly inhibits platelet aggregation induced by thrombin. Inhibiting FX also decreases thrombin generation and thrombus development [9]. AP is a pyrazolopyridine derivative. It is 1-(4-methoxyphenyl)-7-oxo-6-[4-(2-oxopiperidin-1-yl) phenyl]-4,5-dihydropyrazolo [3,4-c]pyridine-3-carboxamide with a molecular weight 459.5 g/mol, chemical formula of C25H25N5O4 [10].

AP is absorbed along with the GIT in the distal part, and the descending colon accounts for 55% of total absorption. It also shows limited dissolution absorption, resulting in slower absorption; C_max_ is achieved in 3–5 h [11]. The bioavailability of AP is around 50 %, with no noticeable effect of food on its absorption [12]. Evidence shows that AP is a substrate of P-gp, and its efflux is affected by P-gp inhibitors like ketoconazole or cyclosporin A on the Caco-cell membrane [13]. AP mainly distributes in extracellular fluids with a volume of 21 L, which is 87–90 % bound to plasma protein, mainly albumin [14]. Elimination of AP involves hepatic metabolism, including O-demethylation, hydroxylation, and sulfation of hydroxylated O-demethyl AP, which occurred primarily via CYP 3A4, with minor contributions from CYP2C9, CYP2C19, CYP1A2, and CYP2C8. It is also excreted into the bile (56%) and in the urine (25 %) as an unchanged drug [15].

Pharmacokinetic drug-drug interaction may occur on any level of pharmacokinetic processes of absorption (especially on P-gp), distribution, and elimination by metabolism or excretion. Omeprazole is a CYP2C19 inhibitor that decreases clopidogrel’s antiplatelet activity, inhibiting the clopidogrel prodrug’s biotransformation into its active metabolite [16]. Its metabolism is affected by CYP2C9 in normal or poor metabolizers [17]. Also, it increases gastric acidity, which might affect the dissolution of another drug with dissolution limited absorption, thus lowering their concentration in the plasma due to incomplete absorption [18]. Apixaban is a P-gp substrate, and it is affected by P-gp inhibitors like ketoconazole, itraconazole, ritonavir, clarithromycin, or verapamil, which increase exposure to AP due to the high amount of drug absorbed; thus, increasing the risk for bleeding [19]. CYP3A4 mainly metabolizes it, and all these enzyme inhibitors would affect its elimination profile in different ways [20]. This study aims to investigate the possibility of any pharmacokinetic interaction between ESO and AP if they were given concomitantly.

## 2 Materials and methods

### 2.1 Chemicals and instruments

ESO magnesium (Sigma) (was given as a gift from Dar-Aldawa Pharmaceuticals in Jordan. AP (batch no-SP-026-144, Sigma), Internal Standard (IS) (0.5 μg AP13C, D3/mL, given by JCPR), acetonitrile and methanol of HPLC gradient grade (Fisher Scientific), ethanol (96%) EMSURE®.

HPLC-MS system with Agilent 1200 series with windows 7, SP1, Vortex Mixer (36 Samples), Labinco, Centrifuge (14,000 rpm), Eppendorf centrifuge 5810 R, Balance Mettler (AT300, Sartorius, five decimals), pH meter (Sartorius 7110), Sonicator (Elmasonic S100), Freezer (General, −20 °C), Water bath BM100 with digital thermostat.

### 2.2 Method development

Samples were analyzed by high-performance liquid chromatography (LC)–triple-quadrupole mass spectrometry using an API 4000™ mass spectrometer (Applied Biosystems/MDS Sciex) coupled to an Agilent 1200 LC system with Windows 7, SP1 as method development and drug analysis for plasma samples [21].

The mobile phase was determined to be a mixture (5 mM ammonium formate: methanol) (25:75%, v/v) with a 650 μL/min flow rate. The column is ACE C8 (50 ∗ 4.6) mm, particle size 5 μm (Supercell), and AP 13C as the internal standard.

#### 2.2.1 Extraction method

The following method was developed and followed to prepare the samples for injection in the instrument:

- 100 μL of blank/spiked plasma was pipetted into a previously labeled tube.
- 30 μL of internal standard (0.5 μg AP 13C, D3/mL) were added and vortexed for 5 s.
- 600 μL of precipitation agent (MeOH) were added and vortexed for 1.0 min.
- Samples were centrifugated for 7 min at 14,000 rpm.
- About 300 μL of the sample was transferred into a glass flat bottom insert's vial and injected.

The chromatographic conditions of Ap and ESO analysis were as follows: The HPLC system: Agilent 1200 series; Detector: API 4000, Applied Biosystems, MDS SCIEX; computer system: Windows 7, SP1; Data Management Software: Analyst 1.6.3; Mobile phase (5 mM Ammonium Formate:Methanol) (25:75%, v/v); Column: ACE C8, (50 ∗ 4.6) mm, particle size 5 μm; Flow Rate: 650 μL/min; Injection Volume: 1 μL; Total run time: 1.60 min; Expected retention time: ESO:1.30 and AP & AP 13C D3:1.10 min.

#### 2.2.2 Method validation

The developed method was tested for both drugs’ matrix effect, linearity, precision, accuracy, and recovery.

Matrix effect was studied by preparing QC Low and QC High solutions, and then six samples of each QC Low and QC High were analyzed. At least 12 blank samples were extracted from at least six different sources (2 blanks for each source). Then, the reconstitution of the extracted samples with prepared QC Low and QC High solutions was followed. Matrix effect was calculated as:

MF of analyte = Peak Area in the presence of Matrix/Peak Area in the absence of Matrix and IS-normalized MF = MF of analyte/MF of IS.

Then, CV% was calculated for the six sources of samples. The acceptance criteria of the ICH guideline is that CV% should be less than 15.00.

The within-run precision of the method was determined by analysis of 6 samples with three replicates, and CV%(s) were calculated.

QC_low_, QC_Mid1,_ QC_Mid2_, and QC_High_ extracted samples of ESO were in concentrations (16 ng/mL), (360 ng/mL), (1200 ng/mL), and QC_High_ (2400 ng/mL) respectively. And for AP, samples; QC_low_ (3 ng/mL), QC_Mid_ 1 (24 ng/mL), QC_Mid__2_ (80 ng/mL) and QC_High_ (160 ng/mL) were extracted.

Linearity of each drug was tested by 8 concentration points (minimum 6 points were required). For ESO (5, 10, 30, 100, 300, 1000, and 3000 ng/mL) and for AP (1, 2, 5, 10, 20, 50, 100, and 200 ng/mL) were used. Correlation coefficient (R) was calculated.

Recovery of the method was tested by analyzing three extracted samples at each level, Low, Mid (Mid-1/Mid-2), and High QC concentrations. Moreover, three extracted blank samples spiked with the analyte postextraction (at low, mid, and high) were analyzed. Recovery was calculated using Eq. (1) below:

(1) $$Recovery\;\%=\frac{Area\;of\;extracted\;plasma\;sample}{Area\;of\;blanks\;spiked\;with\;the\;analyte\;post\;extraction}*100\%$$

% CV was calculated each time.

For ESO: QClow (16 ng/mL), QCMid 1 (360 ng/mL), QCMid 2 (1200 ng/mL), and QCHigh (2400 ng/mL) and for AP, samples; QClow (3 ng/mL), QCMid 1 (24 ng/mL), QCMid 2 (80 ng/mL) and QCHigh (160 ng/mL) were extracted and analyzed.

### 2.3 Preclinical study

The ethical committee approved the study protocol of the High Research Council, Faculty of Pharmacy of the Al-Ahliyya Amman University, and the study was carried out in the animal house of the university. Thirty Wistar rats, eight weeks age, 200 g ± 15 g average weight, were used in this study. They were divided into five groups, each of 6 rats. Group 1 (G1) were given ESO alone as I.V sterile solution (5 mg/kg) in D.W containing 5% ethanol. Group 2 (G2) was given AP as sterile I.V solution (125 mcg/kg) in D.W containing 10% ethanol. Group 3 (G3) were given oral ESO 5 mg/kg as enteric-coated granules freshly suspended in water. Group 4 (G4) was given AP oral solution (250 mcg/kg). Group 5 (G5) was given ESO 5 mg/kg + AP (250 mcg/kg concurrently orally. All rats fasted overnight with access only to water. Blood samples were taken from the tail at the schedule at (0.5, 1, 2, 4, 6, 9, 24, 48) h for oral dosage and intravenous dosing at: (20 min, 40 min, 1 h, 2 h, 5h, 9 h, 24 h, 48 h). Blood samples were drawn into an EDTA tube and immediately centrifuged at 14,000 rpm for 7 min. Plasma was obtained and placed into a labeled Eppendorf tube and stored at −8 °C until analysis.

### 2.4 Pharmacokinetic study

After constructing the Cp vs. t profile by plotting the average plasma concentration of each drug in ng/mL vs. time in hours, the pharmacokinetics was performed using Non-Compartmental Analysis (NCA) using WinNonlin® (Version 8.1). The pharmacokinetic parameters (C_max_, T_max_, AUC_0–24_, AUC_0–∞_″, AUMC_0–24_, AUMC_0–∞_″, MRT0–24, MRT_0–∞_″ Kel (elimination rate constant), t1/2 (elimination half-life), Cl (clearance), V/F (volume of distribution after oral dosing), MAT0–24, MAT_0–∞_″, F were all calculated for ESO and AP alone and in combination (orally) and then compared statistically using Winnolin (version 8.1) software and 10% as Confidence Interval was used.

## 3 Results

### 3.1 Method development and validation

**Figure 1:**
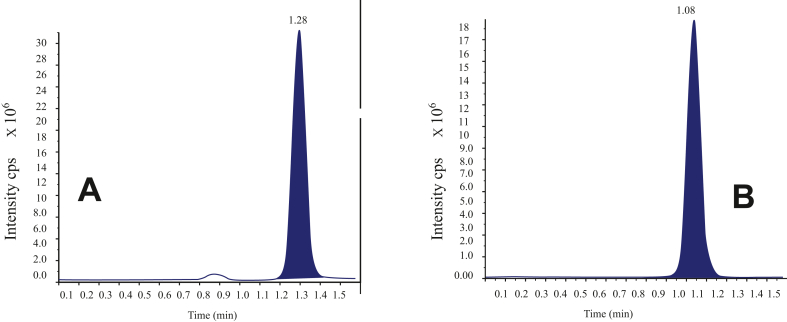
Chromatogram of ESO (A) and AP (B) showing retention time of both peaks.

**Figure 2:**
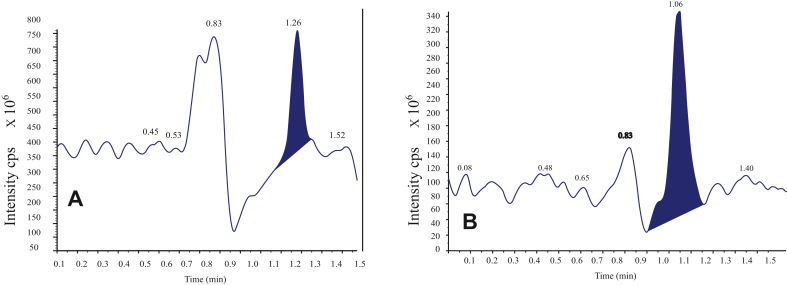
Mass data of (A) ESO and (B) AP.

Figures 1 and 2 show samples of chromatograms of both drugs and mass data. Figure 1(A) shows the chromatogram of ESO with its retention time (RT) of 1.28 min and Figure 1(B) shows the RT of AP of 1.08 min Figure 2(A) and 2(B) show ESO peak of mass data and that of AP respectively. The method was successful in the separation and quantification of both drugs in samples.

Table 1 shows the results of the matrix effect of ESO and AP, showing that CV% was less than 15%, which is the acceptance limit stated by the ICH guideline.

**Table 1:** Results of matrix effect.

IS-Normalized MF (ESO)		
QC Low	0.7054 ± 0.05893	8.35
QC High	0.8250 ± 0.05209	6.31
IS-Normalized MF (AP)		
QC Low	0.9249 ± 0.11312	12.23
QC High	1.0221 ± 0.03586	3.51

The method’s within-run precision and accuracy were determined by analyzing six samples with three replicates (N = 3) on the same day. The CV% was calculated from the standard deviation (SD) ratio to the mean and expressed as a percentage. The acceptable limits of CV%, which should be below 1.5, are obtained for concentration and accuracy. In addition, the accepted accuracy criterium, which was 85–115% for all concentrations, was obtained. Results are shown in Table 2.

**Table 2:** Results of ESO and AP within-day precision and accuracy.

ESO					
Within Run Accuracy					
Sample	LLOQ	QCL	QCM 1	QCM 2	QCH
	5.00 ng/mL	16.00 ng/mL	360.00 ng/mL	1200.00 ng/mL	2400.00 ng/mL
Within Run Precision (ng/mL)					
Mean (measured)	4.061	13.636	370.263	1247.464	2375.308
SD	0.7299	0.3870	17.3300	91.8395	42.3148
CV%	17.97	2.84	4.68	7.36	1.78
Accuracy (%)					
Accuracy	81.22	90.91	102.85	103.96	98.97

AP					
Within Run Precision					
Sample	LLOQ	QCL	QCM 1	QCM 2	QCH
	1.00 ng/mL	3.00 ng/mL	24.00 ng/mL	80.00 ng/mL	160.00 ng/mL
Mean (measured)	1.057	3.004	23.917	82.924	159.692
SD	0.2061	0.4083	1.0594	2.0952	6.5064
CV%	19.50	13.59	4.43	2.53	4.07
Accuracy (%)					
Accuracy	105.70	100.13	99.65	103.66	99.81

The “within the run” accuracy result indicates that the ESO in plasma can be measured with adequate accuracy and precision at all concentration levels. The lower limit of quantitation results shows that the analytical method of ESO can measure the concentrations of those not less than LLOQ accurately and precisely. The same conclusion is obtained from the results of AP.

The linearity of ESO of 8 concentrations between 5.00 ng/mL and 5000 ng/mL gave a linear regression. The linearity determination coefficient R^2^ was 0.999 (R equal to 0.999) as a mean of 3 linearity experiments, which indicates the linearity of the concentration used with the AUC measured for drug and IS. The mean R^2^ of AP calibration concentrations was between 1 ng/mL and 200 ng/mL, repeated three times and 0.997 (mean R = 0.998), meeting the same criteria.

All concentrations of ESO and AP used in the calibration were back-calculated as mean ± SD, and % CV was calculated. Results gave %CV less than 15 % for all samples (2–9 for ESO and 2–7 for AP), which indicated precisely the calibration. Accuracy ranged from 39 % to -106 % for both drugs.

Values of the recovery test applied to QC_low_, QC_mid_, and QC_high_ are within the specifications of ICH guidelines, and the results are shown in Table 4 for both drugs. Recovery of the internal standard ranged between 80% and 91%, with a CV% of 5.72, as shown in Table 3.

**Table 3:** Recovery test results of ESO and AP.

Replicate	QCLow (Peak Area)		QCMed-1 (Peak Area)		QCMed-1 (Peak Area)		QCHigh (Peak Area)	
	Extracted	Post Spiked	Extracted	Post Spiked	Extracted	Post Spiked	Extracted	Post Spiked
ESO								
Mean	5000	4065	116784	103691	353111	330049	644979	616340
Recovery %	123.00		112.63		106.99		104.65	
Mean	111.82							
SD	8.173							
CV%	7.31							
AP								
Mean	1470	1633	9507	12218	30342	40504	63016	74288
Recovery %	90.02		77.81		74.91		84.83	
Mean	81.89							
SD	6.834							
CV%	8.35

**Table 4:** pharmacokinetic parameters of ESO and AP calculated by NCA when given alone and in combination.

pharmacokinetic parameters (unit)	ESO (alone)	ESO (comb.)	AP (alone)	AP (in comb.)	ESO (IV)	AP (IV)
Cp_max_ (ng/mL)	695.9 ± 25.8	387.9 ± 23.5	57.2 ± 5.3	98.6 ± 12.6	–	–
T_max_ (h)	2 ± 0.156	4 ± 0.55	6 ± 1.09	1 ± 0.32	–	–
AUC0–24 (ng.h/mL)	4379.1 ± 218.5	3250.3 ± 160.4	959 ± 38.36	1265.9 ± 63.3	5115.6 ± 120	894.04 ± 80.6
AUC_0–∞_ (ng.h/mL)	4513.5 ± 225.6	3412.3 ± 45.9	1543 ± 54	1560.25 ± 78	5449 ± 118	1344.27 ± 120.5
AUMC0–24 (ng.h^2^/mL)	24658.6 ± 206.5	23544.9 ± 309	9517 ± 475.8	10320.5 ± 516	35805 ± 826	8164.8 ± 78.3
AUMC_0–∞_ (ng.h^2^/mL)	29094.1 ± 1454.7	29430.9 ± 1473	37101 ± 742.2	21710.2 ± 215.1	40322.6 ± 1256	28975.07 ± 2364
MRT∞ (h)	6.4 ± 0.128	8.6 ± **0.13**	24 ± 0.24	14 ± 0.21	7.4 ± 0.95	21.5 ± 1.5
Kel (app) (h^−1^)	0.147 ± 0.005	0.116 **± 0.002**	0.042 ± 0.004	0.0718 ± 0.001	0.142 ± .02	0.0463 ± 0.01
half-life (hr)	4.7 ± 0.14	5.9 ± 0.236	16.6 ± 0.99	9.6 ± 0.144	4.8 ± 0.8	14.9 ± 1.8
Cl (mL/min)	3.7 ± 0.18	4.8 ± 0.14	0.54 ± 0.03	2.6 ± 0.042	3.7 ± 0.7	0.3 ± 0.08
Vd (L)	1.41 ± 0.07	2.4 ± 0.09	0.779 ± 0.032	2.1 ± 0.015	1.5 ± 0.15	0.4 ± 0.09

### 3.2 Pharmacokinetic study

Noncompartmental analysis is widely used in the analysis of pharmacokinetic data. It is based on the calculation of AUC (plasma level-time curve), which represents “drug exposure across time” without considering the distribution pattern of drug distribution. Other pharmacokinetic parameters will be calculated from AUC. The key pharmacokinetic parameter is the elimination rate constant (Kel); the critical pharmacokinetic parameter is calculated from the mean residence time “MRT”. Even though Kel is used in the equations, it is usually only calculated from the curve’s late points.

No nonlinearity was observed when both drugs were given multiple doses during trials. This was significant for PPI (ESO) because it exhibits both linear and nonlinear kinetics, depending on the species’ amount of CYP2C9 and CYP2C19. All curves obtained showed an exponential decline in the elimination phase, indicating linearity.

**Figure 3:**
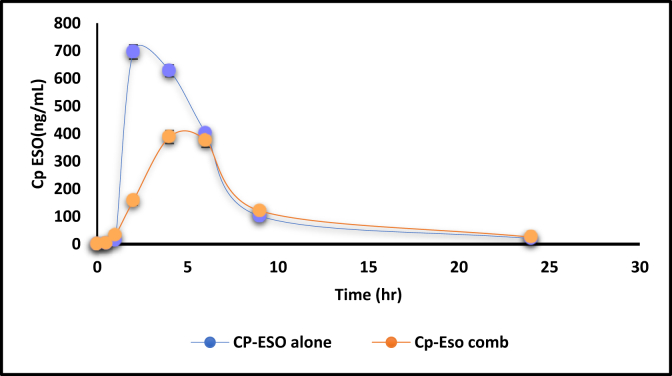
Plasma level-time profile of ESO alone and in combination with AP.

**Figure 4:**
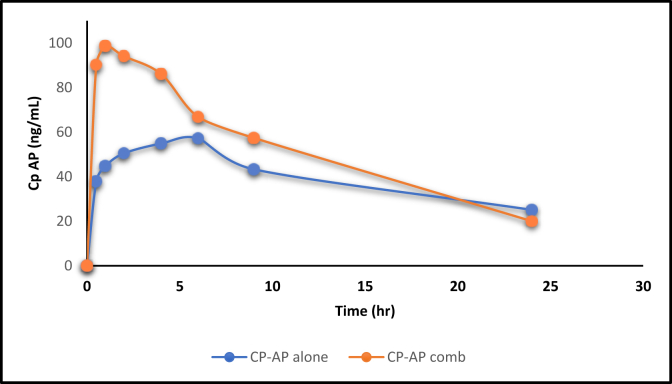
Plasma level-time profile of AP alone and in combination with ESO.

**Figure 5:**
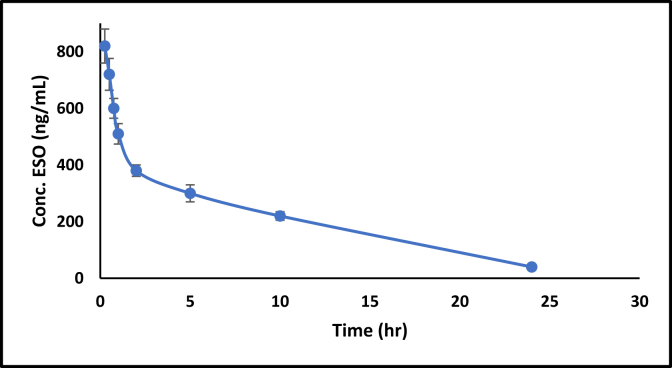
Plasma level-time profile of IV ESO.

Figure 2 shows the plasma level time profile of ESO alone and in combination. Whereas Figure 3 shows the AP when given alone and concurrently with ESO. Figures 4 and 5 show the IV data of ESO and AP, respectively.

Non-compartmental analysis (NCA) was performed using WinNonlin software version 8.1. Pharmacokinetic parameters of both drugs alone and when given together are listed in Table 4.

Figure 3 shows the decrease in C_max_ from 695.9 ± 25.8 to 387.9 ± 23.5 ng/mL and the increase in T_max_ (from 2 h to 4 h) for ESO, which indicates a slower absorption of less amount of ESO when given with AP. The difference is statistically significant, as illustrated in Table 4. Moreover, the total AUC was decreased from 4513.5 ± 225.6 ng h/mL to 3412.3 ± 45.9 ng h/mL, which was also statistically significant.

Calculations of ESO pharmacokinetic parameters alone and in combination with AP revealed an increase in T_max_, a decrease in C_max_, and a decrease in AUC to 24 h and infinity. These basic bioavailability parameters reflect the extent and rate of drug absorption and the activity of elimination processes (AUC represents drug exposure with time). C_max_ is a parameter changing with both extent and rate of drug absorption, and the decision on which one has the primary effect is approved by the change in AUC (extent). Thus, in this case, ESO was absorbed in a lower amount and slower rate when given concurrently with AP.

AUC calculates NCA’s elimination parameters (Kel, half-life, and Cl). That is why when AUC changes, all these parameters will change mathematically, a known drawback of NCA. That is why estimating Kel from the late points in the plot would be more accurate in detecting any real change in the elimination pattern of the drug.

The elimination rate constants of ESO alone and in combination with AP calculated from late concentrations of the plasma level time profile as linear models were 0.163 ± 0.023 h^−1^ and 0.172 ± 0.019 h^−1^, respectively. The calculated Kel at late points in both plots (ESO alone and with AP), where there was no significant change in the elimination rate constant, implying that the elimination pattern of ESO did not change in either case. ESO’s primary route of elimination is through CYP2C9 and 2C19, and this pathway represents a branch pathway of AP metabolism whose main pathway is through CYP3A4.

AUC_0–∞_ results are considered reliable if the AUC extrapolation from 24 to infinity accounts for less than 3% of the total area (up to 20 using this method). The extrapolated area excludes the source of the error, supporting the conclusion that the AUC of ESO changes when given with AP (25 percent decrease). If the results of AP pharmacokinetic parameters calculations give more AP absorbed, this will support some competition for absorbing sites or transporters that permit transporting of AP at the expense of ESO.

The calculations of the AUC of ESO with and without AP are for the calculation of MRT and Ke. The extrapolated area to infinity is about 13% of the total area for ESO alone and 20% of the total area for ESO in combination. Since MRT depends on the difference in area and monumental area and consequently Kel (1/MRT), the large extrapolated area, in this case, could be the reason behind the slight (but significant on 5% CI) change in the elimination rate constant.

For AP, the three primary parameters of drug absorption changed significantly, where C_max_ increased from 57.2 ± 5.3 to 98.6 ± 12.6 (significant) and T_max_ decreased from 6 h to 1 h (significant), suggesting the fast absorption of a higher amount of drug when given in combination (Figure 4 and Table 4). On the other hand, the AUC0-24 h was increased significantly from 959 ± 38.36 to 1265.9 ± 63.3, which means more drug exposure within 24 h was observed when given AP with ESO.

About half of the prescribed doses of AP (2.5 and 5 mg) are known to be bioavailable in humans, with absorption occurring slowly in the gastrointestinal tract. Excluding the factors related to the slow dissolution of solid dosage forms, and because it was given as a solution in this study, the suggested reason is its permeability through the GIT membrane. AP is a P-gp and BCRP (breast cancer receptor protein) substrate subjected to an efflux mechanism [13]. This is an important reason for its low bioavailability. Because ESO is considered a moderate to potent P-gp inhibitor [22], this combination might inhibit the efflux mechanism of AP, which causes an increase in AP absorption, resulting in more drug absorption in a shorter time.

Moreover, the results suggested that the absorption of AP at the expense of ESO may be a kind of competition for absorbing transporters. AP absorption research has also revealed that a reasonable amount of this drug is absorbed by P-gp transporters. The competition here may go towards the benefit of AP [23].

### 3.3 Calculation of bioavailability (F)

**Figure 6:**
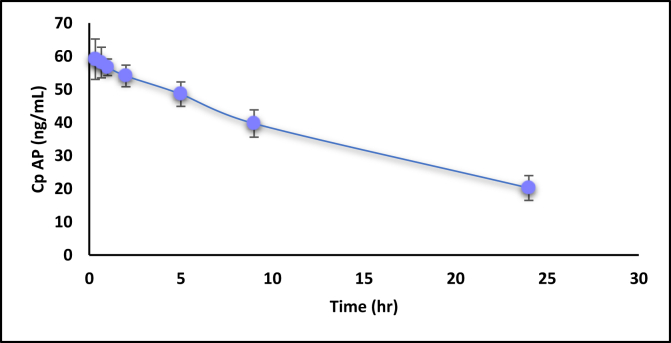
Plasma level-time profile of IV AP.

The absolute bioavailability of both drugs was calculated from oral versus I.V. data using the formula shown in Eq. (2) below:

(2) $$F=\frac{AUC\;oral}{AUC\;iv}∙\frac{Dose\;iv}{Dose\;oral}$$

(F) was calculated when giving each drug alone and when given concurrently. IV data of both drugs are shown in Figures 5 and 6. Results are shown as follows (F) of ESO alone was equal to 0.85∗ ± 0.02 and when given with AP 0.62 ± 0.015 which was significantly less (p < 0.1), while F of AP was equal to 0.42 ± 0.01 when given alone and 0.55∗ ± 0.02 when given with ESO which is significantly higher (p < 0.1).

The results of AP bioavailability calculations based on IV vs. oral data when the drug was given alone and in combination with ESO mentioned above revealed a significant increase in AP bioavailability when given in combination with ESO (data up to 24 h), indicating an increase in the amount of drug absorbed and reaching the systemic circulation. In addition, when combined with AP, ESO bioavailability is reduced.

## 4 Conclusions

Giving AP with ESO concurrently resulted in a decrease in C_max_, AUC of ESO, and an increase in T_max_, suggesting less drug absorbed in a long time. It also resulted in increased C_max_, decreased T_max_, and increased AUC0-24 h of AP, suggesting an increase in the amount of drug absorbed in a shorter time. The bioavailability of AP calculated from IV vs. oral data was increased due to the higher amount of drug absorbed. While the bioavailability of ESO decreased when given with AP.

## Declarations

### Author contribution statement

Ali Jaber: Conceived and designed the experiments; Performed the experiments; Analyzed and interpreted the data.

Israa Al-Ani: Conceived and designed the experiments; Analyzed and interpreted the data; Wrote the paper.

Mohammad Hailat: Conceived and designed the experiments; Performed the experiments; Analyzed and interpreted the data; Contributed reagents, materials, analysis tools or data; Wrote the paper.

Enas Daoud: Contributed reagents, materials, analysis tools or data; Wrote the paper.

Anmar Abu-Rumman: Performed the experiments; Wrote the paper.

Zainab Zakaraya: Performed the experiments; Analyzed and interpreted the data.

Bashar J.M Majeed: Analyzed and interpreted the data; Contributed reagents, materials, analysis tools or data.

Osaid Al Meanazel Performed the experiments; Contributed reagents, materials, analysis tools or data.

Wael Abu Dayyih: Performed the experiments; Contributed reagents, materials, analysis tools or data; Wrote the paper.

### Funding statement

This research did not receive any specific grant from funding agencies in the public, commercial, or not-for-profit sectors.

### Data availability statement

Data included in article/supp. material/referenced in article.

### Declaration of interests statement

The authors declare no conflict of interest.

### Additional information

No additional information is available for this paper.
